# Meningococcal meningitis and complement deficiences

**DOI:** 10.1186/1939-4551-8-S1-A138

**Published:** 2015-04-08

**Authors:** Aline Lury Aoki, Anete Grumach, Fabiane Milena Castro Araújo Pimenta, Vivian Alves Costa, Sandra Mitie Ueda Palma, Michael Kirschfink, Rosemeire Navickas Constantino-Silva, Viviana Arruk, Alexandre Campeas, Maria Do Socorro Ferrão, Myriam Raymundo, Ana Karolinne Burlamaqui Melo

**Affiliations:** 1 Faculty of Medicine ABC, SP, Brazil; 2 University of Heildelberg, Germany; 3 Institute of Infectious Diseases Emilio Ribas, SP, Brazil

## Background

Deficiencies of terminal components of complement have been described in patients affected by meningococcal meningitis. The need of routine investigation has to be established. We evaluated patients with confirmed meningitis due to N. meningitides looking for complement system evaluation.

## Methods

Prospective study in which data and blood samples of patients with confirmed meningococcal meningitis were collected. Hemolytic assays, CH50 and APH50, for classical and alternative pathways respectively, ELISA for properdin and mannose binding lectin (MBL) were performed. Specific components were evaluated after confirmed impairment of complement system.

## Results

A hundred and twenty nine patients (69M:60F) were included in the study. The age of the patients ranged from 2 months (m) up to 64 years old (mean= 96.2m; median=48m). The following serogroups were identified: type C, 36.4%; B, 20.2%; W135, 1.5% and 41.9% had no serogroup identified. CH50 and AP50 values were below the reference levels in 48 patients (37.2%) and 97 patients (75.2%), and the activity was undetectable in 5 and 15 patients, respectively. Levels of CH50 and AP50 were both low in 46 patients (33.65%) and in 8 were both undetectable. Properdin levels were performed in patients with low AP50 (n = 44) and 43.2% had decreased properdin value. MBL values were below 50 micrograms in 2/26 patients evaluated. One patient was diagnosed with C6 deficiency after the second meningitis.

## Conclusions

Although high number of patients had low levels of complement evaluation, it probably represents activation of the system due to meningitis. The study suggests the need of complement evaluation but a period after the acute infection would be more reliable to establish real complement defect.

